# Intracellular and *in vivo* evaluation of imidazo[2,1-*b*]thiazole-5-carboxamide anti-tuberculosis compounds

**DOI:** 10.1371/journal.pone.0227224

**Published:** 2020-01-06

**Authors:** Garrett C. Moraski, Nathalie Deboosère, Kate L. Marshall, Heath A. Weaver, Alexandre Vandeputte, Courtney Hastings, Lisa Woolhiser, Anne J. Lenaerts, Priscille Brodin, Marvin J. Miller

**Affiliations:** 1 Department of Chemistry and Biochemistry, Montana State University, Bozeman, Montana, United States of America; 2 Univ. Lille, CNRS, Inserm, CHU Lille, Institut Pasteur de Lille, U1019 –UMR 8204 –CIIL–Center for Infection and Immunity of Lille, Lille, France; 3 Mycobacteria Research Laboratories, Department of Microbiology, Immunology and Pathology, Colorado State University, Fort Collins, Colorado, United States of America; 4 Department of Chemistry and Biochemistry, University of Notre Dame, Notre Dame, Indiana, United States of America; Institut de Pharmacologie et de Biologie Structurale, FRANCE

## Abstract

The imidazo[2,1-*b*]thiazole-5-carboxamides (ITAs) are a promising class of anti-tuberculosis agents shown to have potent activity *in vitro* and to target QcrB, a key component of the mycobacterial cytochrome bcc-aa3 super complex critical for the electron transport chain. Herein we report the intracellular macrophage potency of nine diverse ITA analogs with MIC values ranging from 0.0625–2.5 μM and mono-drug resistant potency ranging from 0.0017 to 7 μM. The *in vitro* ADME properties (protein binding, CaCo-2, human microsomal stability and CYP450 inhibition) were determined for an outstanding compound of the series, ND-11543. ND-11543 was tolerable at >500 mg/kg in mice and at a dose of 200 mg/kg displayed good drug exposure in mice with an AUC(0-24h) >11,700 ng·hr/mL and a >24 hr half-life. Consistent with the phenotype observed with other QcrB inhibitors, compound ND-11543 showed efficacy in a chronic murine TB infection model when dosed at 200 mg/kg for 4 weeks. The efficacy was not dependent upon exposure, as pre-treatment with a known CYP450-inhibitor did not substantially improve efficacy. The ITAs are an interesting scaffold for the development of new anti-TB drugs especially in combination therapy based on their favorable properties and novel mechanism of action.

## Introduction

**Fig 1:**
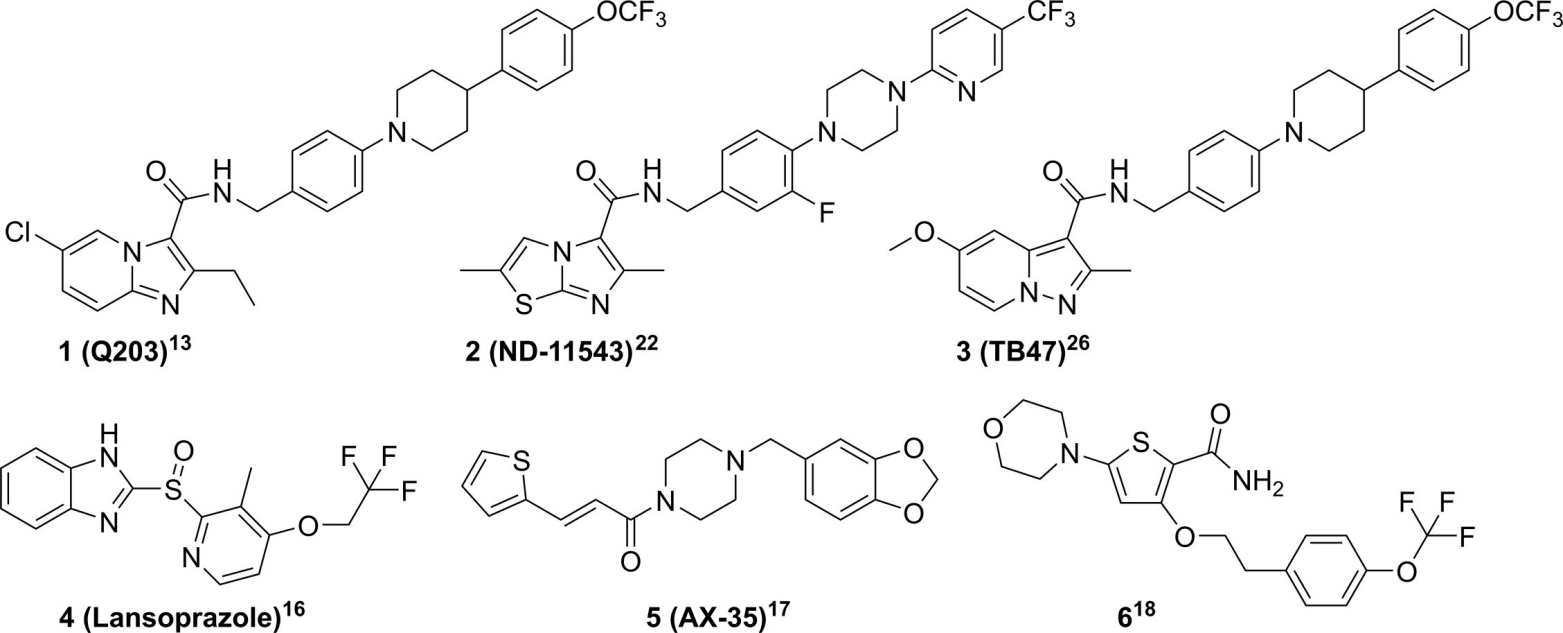
Imidazo[1,2-a]pyridine-3-carboxamides (1), Imidazo[2,1-b]thiazole-5-carboxamides (2), Pyrazolo[1,5-a]pyridine-3-carboxamides (3), Lansoproazole (4), Arylvinylpiperazines (5) and Morpholino Thiophenes (6) are diverse chemical classes that inhibit QcrB.

Tuberculosis (TB) is among the worst diseases to plague humanity. TB kills about 5,000 people each day. As an insidious air borne pathogen, *Mycobacterium tuberculosis*, the causative agent of TB, simultaneously infects millions more each year [1]. The current treatment for drug sensitive TB is a dated combination regimen that includes rifampicin, isoniazid, ethambutol and pyrazinamide, which together cause a myriad of side-effects leading to reduced patient compliance [2]. Despite decades of focused effort to eradicate TB, this disease persists and is becoming more drug resistant. It is estimated that more than 550,000 people have drug resistant TB and only 54% of these people are successfully treated for various reasons, including-economics, drug availability, and lack of effective drugs [3]. It has taken over 40 years for newer agents, such as bedaquiline and pretomanid, to become conditionally approved for the treatment of drug resistant TB [4–6] Bedaquiline validated that targeting energy generation via inhibition of respiratory ATP synthesis is a viable approach, particularly within mycobacteria where respiration is so essential for survival [7]. Our labs have been pursuing new anti-tuberculosis compounds that target energy generation and have discovered and endeavored to optimize the imidazo[1,2-*a*]pyridine-3-carboxamide (IPA) class [8–15]. The IPAs, including Q203 (**1**, Fig 1), target the electron transport chain, specifically at QcrB, a component of the terminal cytochrome oxidase [16, 17]. Various scaffolds have emerged that target QcrB (Fig 1) [16–31], two classes, imidazo[2,1-*b*]thiazole-5-carboxamides (ITAs) (**2**) [26–28] and pyrazolo[1,5-*a*]pyridine-3-carboxamides (**3**) [28–31], bear the greatest structural homology and potency relative to the IPAs (Fig 1). We have disclosed the impressive *in vitro* properties of various ITAs, including low nanomolar potency against replicating and drug-resistant Mtb strains and low cyctotoxicity [26, 27], as exemplified by ND-11543 (**2**). However, the ITAs have not been evaluated *ex vivo* or *in vivo*. The pyrazolo[1,5-*a*]pyridine-3-carboxamides, exemplified by the lead compound TB47 (**3**), have demonstrated low nanomolar potency against replicating *M*. *tuberculosis* and a panel of 56 isolates (including multi- and extensively drug-resistant strains). TB47 itself has MIC values between 0.03–0.93 μM (0.016 and 0.500 μg/mL) [30, 31]. When co-dosed with rifampicin or pyrazinamide, TB47 showed synergistic bactericidal efficacy within the acute murine infection model of tuberculosis and comparable potency to Q203 within the chronic murine infection model [31]. Compounds **4**, **5** and **6** (Fig 1) show some of the diverse chemical structures that can inhibit QcrB. Indeed, the gastric proton pump inhibitor lansoprazole (**4**) undergoes sulfoxide reduction to an active anti-tubercular compound (LPZS) against replicating Mtb (IC_90_ = 1.1–1.7 μM, against three family strains) and drug-resistant Mtb (0.5–1.4 μM, against six strains) as well as efficacy within the acute murine infection model [20]. The arylvinylpiperazines, like AX-35 (**5**), were identified through a large phenotypic screening campaign conducted GlaxoSmithKline. AX-35 has an MIC of 0.14 μM (0.05 μg/mL), has demonstrated good *ex vivo* and *in vivo* efficacy, and is believed to interact with QcrB with a binding mode different than that of Q203 [21]. The morpholino thiophenes represent another point of diversity in structures that can inhibit QcrB. Compound **6** has an MIC of 0.2 μM, overlays well with Q203 by 2D comparison modeling, and has shown efficacy within the acute murine infection model of tuberculosis [22].

In this study, we have selected a focused set of ITAs and evaluated their potency against *M*. *tuberculosis* through *ex vivo* screening in the macrophage, which more closely mimics the physiological conditions of the disease state [32, 33]. Evaluation of ATP synthesis inhibitors within the macrophage, particularly QcrB inhibitors like the ITA class, might be especially informative as this assay takes into consideration the contribution of host cells in eradicating Mtb. Herein we report the microsomal stability and *in vivo* pharmacokinetic properties of an outstanding compound, ND-11543. We also demonstrate that ND-11543 is active when evaluated in the chronic murine model of TB.

## Materials and methods

### Determination of minimum inhibitory concentration (MIC)

MICs were determined in liquid media in 96-well, black, clear-bottom plates as described [14]. A 10-point, 2-fold serial dilution was run for each compound and bacterial growth was measured by OD_590_ after 5 days of incubation at 37°C. Growth inhibition curves were fitted using the Levenberg–Marquardt algorithm [34]. The MIC was defined as the minimum concentration at which growth was completely inhibited and was calculated from the inflection point of the fitted curve to the lower asymptote.

### Mtb non-replicating (low oxygen recovery assay—LORA)

The Low Oxygen Recovery Assay was carried out in 96-well plates as described [35]. Bacteria (*M*. *tuberculosis* strain H37Rv-LUX) were cultured in Dubos media with supplement (DTA) in the Wayne Model of hypoxia [36] for 18 days to enter hypoxia and used to seed 96-well plates containing compounds. Plates were incubated for 9 days under anaerobic conditions followed by 28h outgrowth under aerobic conditions. As a comparator, plates were incubated for 6 days under aerobic conditions. Growth was measured by luminescence. Growth inhibition curves were fitted using the Levenberg–Marquardt algorithm. The MIC was defined as the minimum concentration at which growth was completely inhibited and was calculated from the inflection point of the fitted curve to the lower asymptote.

### Determination of eukaryotic cytotoxicity

Cytotoxicity was tested against the African green monkey adult kidney cell line (VERO, ATCC CRL-1586) as described [37]. Compounds were tested as 10-point, 3-fold serial dilutions in DMSO (final assay concentration of 1% DMSO) and viability was measured using CellTiter-Glo (Promega). Inhibition curves were fitted using the Levenberg–Marquardt algorithm. IC_50_ was defined as the concentration required to reduce cell viability by 50% after 2-day incubation.

### Intracellular mycobacterial replication assay

Raw 264.7 macrophages (ATCC^®^ TIB-71, purchased from American Type Culture Collection) were cultivated in RPMI-1640 Glutamax medium (Gibco) containing 10% heat-inactivated fetal bovine serum (FBS, Gibco; RPMI-FBS). Cells were cultured in a controlled 5% CO_2_ atmosphere at 37°C. A recombinant strain of *M*. *tuberculosis GC1237 of the W/*Beijing type constitutively expressing a green fluorescent protein (Mtb-GFP) was used as a reporter for the intracellular mycobacterial replication assay [38]. This strain was grown in Middlebrook 7H9 media supplemented with 10% oleic acid-albumin-dextrose-catalase (OADC; Difco), 0.05% Tween 80 (Sigma-Aldrich), 0.5% glycerol (Euromedex), and 50 μg/ml of hygromycin B (Invitrogen). Cultures were maintained at 37°C under static conditions for up to 14 days to reach the exponential phase of bacterial growth before being used for the intracellular mycobacterial replication assay.

Raw 264.7 macrophage (4 x 10^5^ cells/mL) suspensions were infected with Mtb-GFP (MOI = 5) in RPMI-FBS for 2 h at 37°C under mild stirring (120 rpm). Cells were washed twice in RPMI-FBS and treated with 50 μg/mL of amikacin (Sigma-Aldrich) for 1 hr at 37°C under mild stirring (120 rpm). The infected cells were washed twice and 50 μL of cell suspension (2 x 10^4^ cells) were added per well in 384-well assay plates (Greiner) containing a 14 point 2-fold dilution series of ITA analog compounds, as well as positive (isoniazid MIC x100) and negative (DMSO 1%) controls. The MICs of tested compounds were assessed in comparison with MICs of two positive controls, BTZ043 (**ND-10379**) and Q203 (**ND-11496**). Microplates were incubated for 5 days at 37°C with 5% CO_2_.

**Fig 2:**
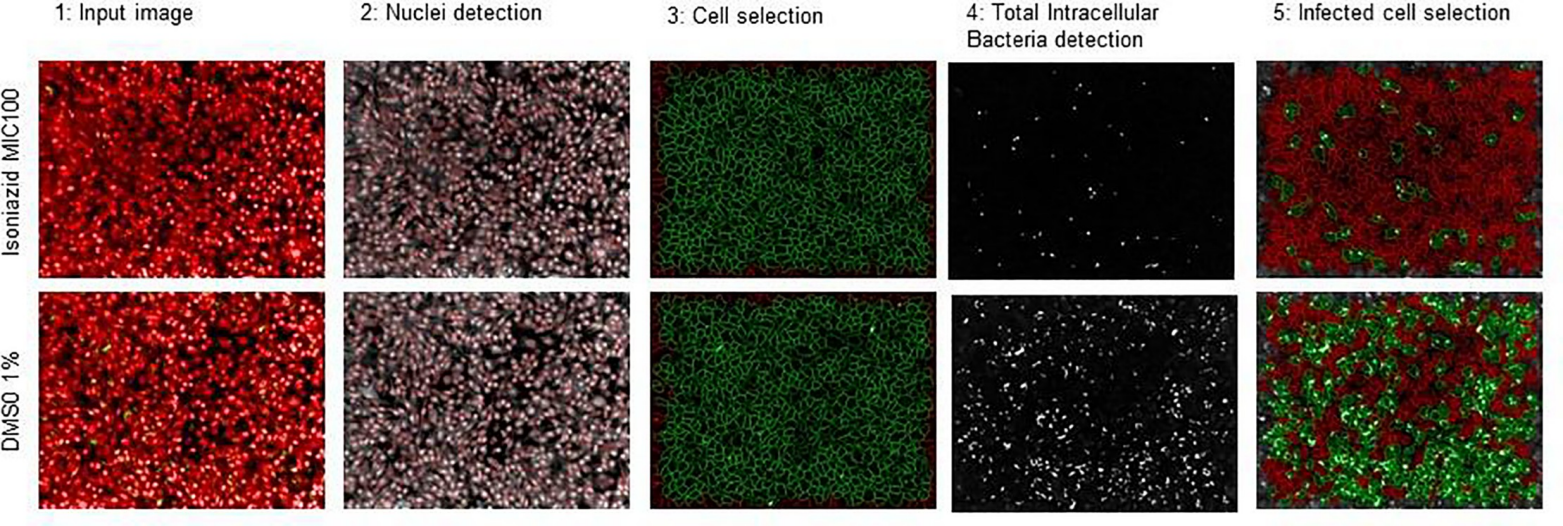
Typical images of controls and the corresponding segmentation for the high content phenotypic infection assay used for the determination of compound effect on *Mycobacterium tuberculosis* intracellular growth inside murine raw 264.7 macrophages. Raw 264.7 macrophages were infected in batch with Mtb-GFP- during 2 hr (MOI = 5) followed by a 1-hr treatment with Amikacin antibiotic to kill the extracellular mycobacteria. The infected cells were then added to 384-well assay plates containing a 14 serial 10-fold dilutions of ITA analog compounds, as well as positive (Isoniazid (INH) 10 μg/mL MIC100) and negative (DMSO 1%) controls. Next, plates were incubated for 5 days at 37°C under a CO_2_ atmosphere. After macrophage labeling with Syto60, images of macrophages infected with Mtb-GFP were acquired by automated confocal microscopy (OPERA^™^ High Content Screening System (PerkinElmer)) and a multiparametric quantification of the infection was performed using automated image analysis. Cells (nuclei and cytoplasm) were detected by an intensity detection algorithm applied on the Syto60 channel. A spot detection algorithm based on the GFP channel was applied for the detection of *Mtb*-GFP in cells and the bacterial intensity and area in pixels were measured. The bacterial growth was quantified by the total intracellular bacterial area (pixel) per well.

Macrophages were then stained with 5 μM Syto60 (Invitrogen) for 30 min at 37°C with 5% CO_2_ before imaging. Image acquisitions were performed on an automated confocal microscope (PerkinElmer) using a 20X water objective. GFP-bacteria and Syto60 signal were detected using 488 and 630 nm laser excitation with a 540/75 and 690/70 nm emission filter, respectively. A series of six images were taken by well, and dedicated image-based analyses were performed using Columbus software (version 2.5.1, PerkinElmer) (Fig 2 and S1 Table in Supplementary data) [32, 33]. Briefly, the two images were first segmented to remove background and to normalize pixel intensities between images. Then, cell nuclei and cytoplasm were detected using an intensity detection algorithm applied on the Syto60 channel. Properties of intensity and morphology were filtered to correctly select the cell population. A spot detection algorithm based on the GFP channel was applied for the detection of Mtb-GFP in cells. The bacterial intensity and area in pixels were measured. The image-based parameters that correlate to bacterial growth are the percentage of infected cells and the bacterial area (pixel) per cell and per well. The number of cells informed on the cytotoxicity of the compound for effective concentrations. Typically, drugs demonstrated a dose-dependent decrease on both bacterial area and percentage of infected macrophages. In this study, the parameter used as a read-out was the area of bacteria present per cell and per well (expressed in px) for infected Raw264.7. Normalizations were performed based on the average values obtained for the negative (DMSO 1%) and positive (isoniazid 10 μg/mL) controls. Inhibition of bacterial replication was determined for each compound. Percentage of inhibition effect was plotted against the log10 of compound concentrations. Fitting was performed by Prism software using the sigmoidal dose-response (variable slope) model. The minimum inhibitory concentration (MIC) was determined and corresponded to the first concentration on the top of the curve, corresponding to the obtained maximum inhibition. Cell viability was measured by comparison of the cell number obtained for each compound concentration to the average value obtained for the positive control (isoniazid 10 μg/mL). Data of two replicates were averaged.

1: Typical 2-color image; 2 and 3: Circled objects correspond respectively to detected nuclei and detected cells, 4: Filled white objects correspond respectively to total intracellular bacteria area, 5: Circled green cells correspond to infected cells.

### Solubility determination

The relative solubility of compounds was determined in microbiological medium using turbidity as a measure [27]. A serial dilution of compounds was prepared in DMSO and then transferred to microbiological medium at pH 6.8. Turbidity was measured and compared to a control (diethylstilbestrol, 125 μM); the lowest concentration at which compounds were insoluble (defined as turbidity >300% of control) was recorded.

### Plasma protein binding

Plasma protein binding for each test compound was determined by equilibrium dialysis [27]. Compounds were tested using a semi-permeable membrane which separates two compartments containing protein (human plasma) and buffer. Molecules can penetrate freely, but proteins cannot pass through the membrane. Test compounds were mixed with human plasma and applied to the device; after equilibration at 37°C with PBS, the test compound in each compartment was quantified by LC-MS/MS. Human pooled plasma used in this experiment was purchased from Bioreclamation Inc. (catalog number HMPLNAHP).

### Caco-2

The permeability of test compounds was assessed using a Caco-2 cell monolayer [27]. Compound permeability was measured in both directions. For A-B permeability, test compound was added to the apical side of the Caco-2 monolayer and the transport of compound to the basal side was monitored. For B-A permeability, test compound was added to the basal side of the Caco-2 monolayer and the transport of the compound to the apical side was monitored. Assays were run for 2h in duplicate. The amount of compound present in each compartment was quantified by LC-MS/MS. The Caco-2 cell line (ATCC HTB-3) used in this experiment was purchased from American Type Culture Collection.

### Cytochrome P450 enzyme inhibition

Compounds were tested for inhibition of six cytochrome P450 enzyme isoforms—CYP2B6, CYP2C8, CYP2C9, CYP2C19, CYP2D6 and CYP3A4 [27]. For each assay, human liver microsomes were incubated with a probe substrate for each CYP isoform in the presence of compound. Compounds were prepared as a 7-point dilution series in acetonitrile:DMSO (9:1). The final DMSO content in the reaction mixture was equal in all solutions used within an assay and was < 0.2%. Samples were run in duplicate.

### Human microsomal stability

Compounds were tested for microsomal stability using pooled human liver S9 microsomes [27]. Microsomes were incubated with the test compound at 37°C in the presence of the co-factor NADPH. The reaction was terminated, the supernatant recovered, and test compounds quantified by LC-MS/MS. A fixed concentration of test compound was tested in duplicate at 5 timepoints and compound stability was expressed as a function of time. Human liver microsomes were purchased from Celsis (catalog number X008068).

### Animal welfare and procedures

All animal studies were performed at Colorado State University in a certified bio-safety level III animal facility in strict accordance to the regulations and recommendations of the Guide for the Care and Use of Laboratory Animals of the National Institutes of Health and the Centers of Disease Control. All procedures and performed protocols for infecting mice with *M*. *tuberculosis* and subsequent drug treatments in the described mouse infection studies were approved by the Colorado State University Institutional Animal Care and Use Committee (IACUC) (Reference numbers of approved protocols: 09-1367A, 12-3723A, 15-5942A, 18-8006A). All experiments were approved by IACUC prior to initiation of the mouse studies and conducted following the relevant guidelines and regulations. Mice were euthanized by CO_2_ inhalation, a method approved by the CSU IACUC.

When animals are infected with *M*. *tuberculosis*, there is no physical discomfort when inside the aerosol machine. The animals can move freely inside large wire baskets, and this process is brief [less than 25 minutes]. The veterinarians at the Laboratory Animal Resources at Colorado State University have procedures in place to control animal pain, as defined by the Karnofski scale. The parameters used to monitor pain and severity of disease of *M*. *tuberculosis* infected mice are as follows: 1) parameters monitored daily to determine the condition of the mice (activity and temperament, feeding behavior, appearance), 2) parameters, which when evident, require that the mice be humanely euthanized within the day (severe physiologic changes, 20% weight loss). Analgesics, anesthesia, or sedation

#### *In vivo* safety study

In this acute toxicity test, 3 healthy female mice (6–8 week old Balb/c from Charles River) were given three consecutive daily doses of ND-11543 and were observed at regular times for any adverse effects. ND-11543 was dissolved in 20% TPGS (d-α tocopheryl polyethylene glycol 1000 succinate) to give a fine white suspension which was dosed at 100, 300 and 500 mg/kg orally on three consecutive days, 3 mice per dose group. Mice were observed 10 min, 1, 2, 4 hr after dosing and then daily afterwards. During the observations, humane handling practices and animal welfare regulations were strictly followed.

#### Single dose plasma PK

Basic pharmacokinetic analysis was determined after a single dose of 200 mg/kg with and without the administration of 100 mg/kg of 1-aminobenzotriazole (ABT) (Sigma, #A3940) given 2 hr by gavage prior to ND-11543 administration. ABT (100 mg/kg dose) was administered by oral gavage in 0.5% (w/v) methyl cellulose 400 solution. ND-11543 (200 mg/kg dose) was prepared in 20% TPGS and dosed orally 2 hr after ABT administration. Female mice (6–8 week old Balb/c from Charles River) were rested one to two weeks before dosing. Blood was collected from the mice at time points of 0.5, 2, 4, 8, 12 and 24 hr after drug administration. Blood samples (< 100 μL each) for all time points were collected via the mandibular vein in plasma collector tubes, centrifuged and plasma (< 50 μL each) was frozen until further use. Plasma samples were analyzed by LC/MS to determine drug plasma levels.

#### *In vivo* chronic Balb/c model of TB infection

A chronic Balb/c infection model with *M*. *tuberculosis* Erdman was carried out as previously described [39]. Female Balb/c mice, aged 6−8 weeks (from Charles River), were infected by the aerosol route on day 0 using the inhalation exposure system (Glas-col Inc., Terre Haute, IN) to give a lung bacterial load of an average of ~50–100 colony forming units (CFU) per mouse. Three mice were sacrificed day 1 post-infection to determine bacterial uptake. Whole lungs were extracted, homogenized in PBS buffer and plated on 7H11/OADC agar plates. The plates were placed in a 37^o^ C dry-air incubator for ~3 weeks. At day 28 post-infection, 5 mice were sacrificed to determine bacterial load in the lungs and spleens at the start of therapy. Lung and spleen homogenate homogenized in PBS buffer were plated on 7H11/OADC agar plates. Each compound was administered via oral gavage on day 28 post-infection with and without the administration of 100 mg/kg of 1- aminobenzotriazole ABT (Sigma, #A3940) given 2 hr prior to test dose and continued five days a week for 30 days until day 58. A control group, isoniazid (INH), was administered via oral gavage at 25 mg/Kg/day, at 200 μL/mouse. Day 58 post-infection, 5 mice of each group were sacrificed, and bacterial loads were determined. Lung and spleen homogenate in PBS buffer were plated on 7H11/OADC agar plates. Statistical analysis was performed by first converting CFU to logarithms, which were then evaluated by a one-way ANOVA followed by a multiple comparison analysis of variance by a one-way Tukey test (SigmaStat software program). Differences were considered significant at the 95% level of confidence.

### Chemical syntheses

The syntheses and characterization of imidazo[2,1-*b*]thiazole-5-carboxamide (ITA) ND-11543 has been previously reported [26] and was profiled in many additional assays described herein.

#### Chemistry

All anhydrous solvents, reagent grade solvents for chromatography and starting materials were purchased from either Aldrich Chemical Co. (Milwaulkee, WI) or Fisher Scientific (Suwanee, GA) unless otherwise noted. Water was distilled and purified through a Milli-Q water system (Millipore Corp., Bedford, MA). General methods of purification of compounds involved the use of silica cartridges purchased from Practichem, LLC. (www.practicachem.com) and/or recrystallization. The reactions were monitored by TLC on precoated Merck 60 F254 silica gel plates and visualized using UV light (254 nm). All compounds were analyzed for purity by HPLC and characterized by ^1^H and ^13^C NMR using a Bruker DPX Avance I NMR Spectrometer (300MHz) and/or a Bruker Ascend Avance III HD Spectrometer (500 MHz). Chemical shifts are reported in ppm (δ) relative to the residual solvent peak in the corresponding spectra; chloroform δ 7.27 and δ 77.23, methanol δ 3.31 and δ 49.00 and coupling constants (*J*) are reported in hertz (Hz) (where, s = singlet, bs = broad singlet, d = doublet, dd = double doublet, bd = broad doublet, ddd = double doublet of doublet, t = triplet, tt–triple triplet, q = quartet, m = multiplet) and analyzed using ACD NMR data processing. ^19^F NMR were run without a standard and are uncorrected. Mass spectra values are reported as m/z. Melting points were measured on a Thomas-Hoover capillary melting point apparatus and are uncorrected.

#### LC-MS method

Liquid Chromatography-Mass Spectrometry method was performed on an Agilent 1290 infinity coupled to Agilent 6538 Ultra High Definition Quadrupole Time of Flight (UHD-QToF) instrument. A separation was achieved by using reverse phase Waters Acquity UPLC HSS T3 1.8μm (2.1 X 100mm) column from Waters (Milford, USA). All solvents were purchased from Fischer Scientific LCMS Optima grade solvents. Water containing 0.1% formic acid was used as mobile phase A and acetonitrile containing 0.1% formic acid was used as mobile phase B. The injection volume was set at 1 μL. Samples were injected in a gradient of 95% mobile phase A and 5% mobile phase B in the initial condition to 5% mobile phase A and 95% mobile phase B in 9 min. The eluent was held at that composition for an additional 3 min and switched back to the initial condition at 12 min.

The MS data acquisition was performed from 50-1000m/z at 1.0 spectra/sec scan rate. The source gas temperature was set at 350°C with a flow of 8 l/min. The nebulizer gas was set at 55 psig. The capillary voltage was set at 3500 volts with fragmentor at 100, skimmer at 45 and octopole RF 500 volts. Prior to sample runs, the instrument was calibrated using Agilent low mass calibrant solution.

#### Data analysis

The data collected in Agilent LC-MS was analyzed using Agilent Mass Hunter software for HRMS calculation.

#### Representative example of EDC-mediated coupling used to prepare analogs

Synthesis of *N*-(3-Fluoro-4-(4-(5-(trifluoromethyl)pyridin-2-yl)piperazin-1-yl)benzyl)-2,6-dimethylimidazo[2,1-*b*]thiazole-5 carboxamide (ND-11543) [26].

2,6-Dimethylimidazo[2,1-*b*]thiazole-5-carboxylic acid (CAS #: 1007875-19-5, 1.17 g, 5.9 mmol) and (3-fluoro-4-(4-(5-(trifluoromethyl)pyridin-2-yl)piperazin-1-yl)phenyl)methanamine (CAS #: 1897429-40-1, 2.29 g, 7.2 mmol) were dissolved in dry CH_3_CN. EDC-HCl (1.38 g, 7.2 mmol) and 4-(dimethylamino)pyridine (DMAP, 965 mg, 7.2 mmol) were added and the reaction was stirred for 12 h at room temperature. The reaction was concentrated *in vacuo*. The residue was taken up in CH_2_Cl_2_ and washed with 5% aqueous acetic acid (2x), saturated aqueous NaHCO_3_ (2x), brine and then dried over sodium sulfate. The drying agent was removed by filtration and the organic layer was concentrated *in vacuo*. The resulting residue was purified by recrystallization with hot CH_3_CN to give *N*-(3-fluoro-4-(4-(5-(trifluoromethyl)pyridin-2-yl)piperazin-1-yl)benzyl)-2,6-dimethylimidazo[2,1-*b*]thiazole-5-carboxamide (ND-11543) as an off white solid, 2.61 g (62% yield). ^1^H NMR (500 MHz, CDCl_3_) δ ppm 8.40 (d, *J* = 2.5 Hz, 1H), 7.99–7.95 (m, 1H), 7.63 (dd, *J* = 9.0, 2.6 Hz, 1H), 7.09–7.03 (m, 2H), 6.92 (t, *J* = 8.4 Hz, 1H), 6.67 (d, *J* = 9.0 Hz, 1H), 5.99 (bt, *J* = 5.8 Hz, 1H, NH), 4.57 (d, *J* = 5.8 Hz, 2H), 3.79 (t, *J* = 5.0 Hz, 4H), 3.15 (t, *J* = 5.0 Hz, 4H), 2.56 (s, 3H), 2.41 (s, 3H). ^13^C (126 MHz, CDCl_3_) δ ppm 160.43, 160.30, 156.69, 154.72, 145.71 (aq, *J* = 4.0 Hz), 144.01, 139.12 (d, *J* = 8.6 Hz), 134.57 (d, *J* = 2.6 Hz), 134.38 (d, *J* = 7.1 Hz), 126.75, 123.53 (d, *J* = 2.7 Hz), 122.33 (q, *J* = 271.6 Hz), 119.28 (d, *J* = 2.7 Hz), 118.10, 115.65, 115.52 (q, *J* = 33.4 Hz), 115.49, 105.64, 50.29 (d, *J* = 2.4 Hz), 44.84, 42.56, 16.48, 13.93. ^19^F NMR (282 MHz, CDCl_3_) δ ppm -61.12 (s, 3F), -121.84 (dd, *J* = 12.9, 8.7 Hz, 1F). HRMS (EI), M + 1 calcd. for C_25_H_25_F_4_N_6_OS, 533.1759; found 533.1741. HPLC t_R_ = 5.2–5.3 min, mp = 234–235 ^o^C.

*N*-((2,3-Dihydrobenzofuran-5-yl)methyl)-2,6-dimethylimidazo[2,1-*b*]thiazole-5-carboxamide (ND-11459) was prepared by the method described for ND-11543 except 5-(aminomethyl)-2,3-dihydrobenzofuran hydrochloride (CAS #: 635309-62-5) was used.

^1^H NMR (500 MHz, CDCl_3_) δ ppm 8.00 (q, *J =* 1.2 Hz, 1H), 7.21 (s, 1H), 7.09 (d, *J* = 8.2 Hz, 1H), 6.76 (d, *J* = 8.2 Hz, 1H), 5.92 (bt, *J* = 6.0 Hz, 1H, NH), 4.57 (t, *J* = 8.6 Hz, 2H), 4.56 (d, *J* = 6.3 Hz, 2H), 3.20 (t, *J* = 8.9 Hz, 2H), 2.55 (s, 3H), 2.44 (d, *J* = 1.5 Hz, 3H). ^13^C NMR (125 MHz, CDCl_3_) δ ppm 160.37, 159.70, 150.38, 143.94, 130.23, 127.71, 127.62, 126.51, 124.59, 118.22, 118.12, 109.35, 71.34, 43.24, 29.65, 16.43, 13.91. HRMS (EI), M + 1 calcd. for C_17_H_18_N_3_O_2_S, 328.1120; found 328.1105. HPLC t_R_ = 6.3 min, mp = 129–130°C.

*N*-((2,3-Dihydrobenzofuran-5-yl)methyl)-6-ethyl-2-methylimidazo[2,1-*b*]thiazole-5-carboxamide (ND-11503) was prepared by the method described for ND-11543 except 5-(aminomethyl)-2,3-dihydrobenzofuran hydrochloride (CAS #: 635309-62-5) was used.

^1^H NMR (600 MHz, CDCl_3_) δ ppm 8.01 (s, 1H), 7.20 (s, 1H), 7.09 (d, *J* = 7.7 Hz, 1H), 6.76 (d, *J* = 8.5 Hz), 5.93 (bt, *J* = 6.2 Hz, 1H, NH), 4.58 (t, *J* = 8.8 Hz, 2H), 4.57 (d, *J* = 5.2 Hz, 2H), 3.21 (t, *J* = 8.4 Hz, 2H), 2.85 (q, *J* = 7.7 Hz, 2H), 2.44 (s, 3H), 1.35 (t, *J* = 7.7 Hz, 3H). ^13^C NMR (151 MHz, CDCl_3_) δ ppm 160.38, 159.71, 150.50, 149.64, 130.18, 127.72, 127.59, 126.59, 124.57, 118.14, 117.49, 109.36, 71.35, 43.27, 29.65, 23.42, 13.95, 13.35. HRMS (EI), M + 1 calcd. for C_18_H_20_N_3_O_2_S, 342.1276; found 342.1269. HPLC t_R_ = 6.8 min, mp = 108–109°C.

2,6-Dimethyl-*N*-(4-(3-(trifluoromethyl)phenoxy)benzyl)imidazo[2,1-*b*]thiazole-5-carboxamide (ND-11564) was prepared by the method described for ND-11543 except [4-[(3-trifluoromethylphenyl)oxy]benzyl]amine (CAS #: 864263-13-8) was used.

^1^H NMR (500 MHz, CDCl_3_) δ ppm 8.00 (q, *J* = 1.2 Hz, 1H), 7.44 (dd, *J* = 7.7, 7.7 Hz, 1H), 7.40–7.34 (m, 3H), 7.25 (dd, *J* = 1.8, 1.8 Hz, 1H), 7.04–7.00 (m, 2H), 6.04 (bt, *J* = 5.5 Hz, 1H, NH), 4.67 (d, *J* = 5.8 Hz, 2H), 2.60 (s, 3H), 2.44 (d, 1.5 Hz, 3H). ^13^C NMR (125 MHz, CDCl_3_) δ ppm 160.49, 157.65, 155.66, 150.51, 144.04, 134.16, 132.27 (q, *J* = 33.3 Hz), 130.32, 129.57, 126.71, 123.56 (q, *J* = 273 Hz), 121.64, 119.78 (q, *J* = 3.1 Hz), 119.65, 118.12, 115.31 (q, *J* = 3.9 Hz), 42.81, 16.48, 13.92. ^19^F NMR (282 MHz, CDCl_3_) δ ppm -66.66 (s, 3H). HRMS (EI), M + 1 calcd. For C_22_H_19_F_3_N_3_O_2_S, 446.1150; found 446.1136. HPLC t_R_ = 8.6 min, mp = 103–104°C.

2,6-Dimethyl-*N*-(4-(3-(trifluoromethyl)phenoxy)phenyl)imidazo[2,1-*b*]thiazole-5-carboxamide (ND-11566) was prepared by the method described for ND-11543 except 4-(3-(trifluoromethyl)phenoxy)aniline (CAS #: 41605-31-6) was used.

^1^H NMR (600 MHz, CDCl_3_) δ ppm 7.99 (s, 1H), 7.60 (d, *J* = 8.8 Hz, 2H), 7.47 (s, 1H), 7.44 (t, *J* = 8.0 Hz, 1H), 7.34 (d, *J* = 7.7 Hz, 1H), 7.24 (s, 1H), 7.17 (d, *J* = 8.4 Hz), 7.07 (d, *J* = 8.8 Hz, 2H), 2.73 (s, 3H), 2.46 (s, 3H). ^13^C NMR (151 MHz, CDCl_3_) δ ppm 158.54, 158.12, 152.55, 151.09, 144.46, 133.80, 132.26 (q, *J* = 33.8 Hz), 130.32, 127.14, 124.59 (q, *J* = 272 Hz), 122.24, 121.19, 120.40, 119.56 (q, *J* = 4.4 Hz), 118.32, 118.07, 114.83 (q, *J* = 3.3 Hz), 16.61, 13.98. ^19^F NMR (282 MHz, CDCl_3_) δ ppm -62.65 (s, 3F). HRMS (EI), M + 1 calcd. For C_21_H_17_F_3_N_3_O_2_S, 432.0994; found 432.0974. HPLC t_R_ = 8.9 min, mp = 149–150°C.

*N*-(4-(1,1-Dioxidothiomorpholino)-3-fluorobenzyl)-2,6-dimethylimidazo[2,1-*b*]thiazole-5-carboxamide (ND-11903) was prepared by the method described for ND-11543 except 4-(4-(aminomethyl)-2-fluorophenyl)thiomorpholine 1,1-dioxide (CAS #: 1155537-01-1) was used.

^1^H NMR (500 MHz, CDCl_3_) δ ppm 7.98 (q, *J* = 1.6 Hz, 1H), 7.11–7.07 (m, 2H), 6.96 (dd, *J* = 8.3, 8.3 Hz, 1H), 6.04 (bt, *J* = 5.5 Hz, 1H, NH), 4.59 (d, *J* = 6.1 Hz, 2H), 3.62–3.59 (m, 4H), 3.23–3.18 (m, 4H), 2.59 (s, 3H), 2.44 (d, *J* = 1.3 Hz, 3H). ^13^C NMR (125 MHz, CDCl_3_) δ ppm 160.49, 153.62 (d, *J* = 247.0 Hz), 150.60, 144.13, 137.62 (d, *J* = 10.0 Hz), 135.03 (d, *J* = 7.5 Hz), 126.80, 123.57 (d, *J* = 3.7 Hz), 120.84 (d, *J* = 2.7 Hz), 118.08, 117.99, 115.80 (d, *J* = 23.1 Hz). ^19^F NMR (282 MHz, CDCl_3_) δ ppm -121.90 (dd, *J* = 12.2, 9.2 Hz, 1F). HRMS (EI), M + 1 calcd. For C_19_H_22_FN4_3_O_3_S_2_, 437.1117; found 437.1099. HPLC t_R_ = 5.98 min, mp = 230–232°C.

*N*-(4-(1,1-Dioxidothiomorpholino)benzyl)-2,6-dimethylimidazo[2,1-*b*]thiazole-5-carboxamide (ND-12015) was prepared by the method described for ND-11543 except 4-(4-(aminomethyl)phenyl)thiomorpholine 1,1-dioxide (CAS #: 1152880-11-9) was used.

^1^H NMR (600 MHz, CDCl_3_) δ ppm 8.00 (s, 1H), 7.31 (d, *J* = 8.4 Hz, 2H), 6.91 (d, *J* = 8.8 Hz, 2H), 5.96 (bt, *J* = 5.0 Hz, 1H, NH), 4.59 (d, *J* = 5.5 Hz, 2H), 3.86 (dd, *J* = 4.7, 4.7 Hz, 4H), 3.11 (dd, *J* = 5.2, 5.2 Hz, 4H), 2.58 (s, 3H), 2.44 (s, 3H). ^13^C NMR (151 MHz, CDCl_3_) δ ppm 160.41, 150.39, 147.05, 143.85, 130.57, 129.23, 126.73, 118.16, 118.11, 116.58, 50.46, 47.64, 42.77, 16.44, 13.92. HRMS (EI), M + 1 calcd. For C_19_H_23_N_4_O_3_S_2_, 419.1212; found 419.1203. HPLC t_R_ = 5.6 min, mp = 214–215°C.

6-Ethyl-2-methyl-*N*-((6-(3-(trifluoromethyl)phenoxy)yridine-3-yl)methyl)imidazo[2,1-*b*]thiazole-5-carboxamide (ND-12024) was prepared by the method described for ND-11543 except (6-(3-(trifluoromethyl)phenoxy)yridine-3-yl)methanamine (CAS #: 197459-39-5) was used.

^1^H NMR (500 MHz, CDCl_3_) δ ppm 8.18 (s, 1H), 7.98 (d, *J* = 1.3 Hz, 1H), 7.79 (dd, *J* = 8.6, 2.2 Hz, 1H), 7.52 (dd, *J* = 7.9, 7.9 Hz, 1H), 7.46 (d, *J* = 7.7, 1H), 7.41 (s, 1H), 7.33 (d, *J* = 7.7 Hz, 1H), 6.98 (d, *J* = 8.6 Hz, 1H), 6.08 (bt, *J* = 5.5 Hz, 1H, NH), 4.64 (d, *J* = 5.8 Hz, 2H), 2.88 (q, *J* = 7.7 Hz, 2H), 2.44 (s, 3H), 1.38 (t, *J* = 7.7 Hz, 3H). ^13^C NMR (125 MHz, CDCl_3_) δ ppm 162.55, 160.58, 154.22, 150.77, 149.94, 146.67, 139.65, 132.08 (q, *J* = 35.4 Hz), 130.11, 129.45, 126.91, 124.54, 123.69 (q, *J* = 270 Hz), 121.35 (q, *J* = 5.6 Hz), 118.24 (q, *J* = 3.1 Hz), 118.06, 117.19, 112.04, 40.31, 23.51, 13.93, 13.35. ^19^F NMR (282 MHz, CDCl_3_) δ ppm -62.59 (s, 3F). HRMS (EI), M + 1 calcd. For C_22_H_20_F_3_N_4_O_2_S, 461.1259; found 461.1250. HPLC t_R_ = 8.3 min, mp = 104–106°C.

2,6-Dimethyl-*N*-((6-(3-(trifluoromethyl)phenoxy)yridine-3-yl)methyl)imidazo[2,1-*b*]thiazole-5-carboxamide (ND-12025) was prepared by the method described for ND-11543 except (6-(3-(trifluoromethyl)phenoxy)pyridine-3-yl)methanamine (CAS #: 197459-39-5) was used.

^1^H NMR (600 MHz, CDCl_3_) δ ppm 8.18 (s, 1H), 7.98 (s, 1H), 7.80 (dd, *J* = 8.5, 2.2 Hz, 1H), 7.51 (t, *J* = 8.0 Hz, 1H), 7.46 (d, *J* = 7.7 Hz, 1H), 7.41 (s, 1H), 7.33 (d, *J* = 8.0 Hz, 1H), 6.97 (d, *J* = 8.5 Hz, 1H), 6.07 (bt, *J* = 5.4 Hz, 1H, NH), 4.63 (d, *J* = 5.9 Hz, 2H), 2.59 (s, 3H), 2.45 (s, 3H). ^13^C NMR (151 MHz, CDCl_3_) δ ppm 162.56, 160.55, 154.21, 150.61, 146.69, 144.17, 139.68, 132.03 (q, *J* = 31.3 Hz), 130.11, 129.47, 126.89, 123.71 (q, *J* = 272 Hz), 124.55, 121.36 (q, *J* = 3.3 Hz), 118.25 (q, *J* = 4.5 Hz), 118.05, 117.95, 112.04, 40.28, 116.49, 13.92. ^19^F NMR (282 MHz, CDCl_3_) δ ppm -62.59 (s, 3F). HRMS (EI), M + 1 calcd. For C_21_H_18_F_3_N_4_O_2_S, 447.1103; found 447.1091. HPLC t_R_ = 7.9 min, mp = 105–106°C.

## Results and discussion

**Fig 3:**
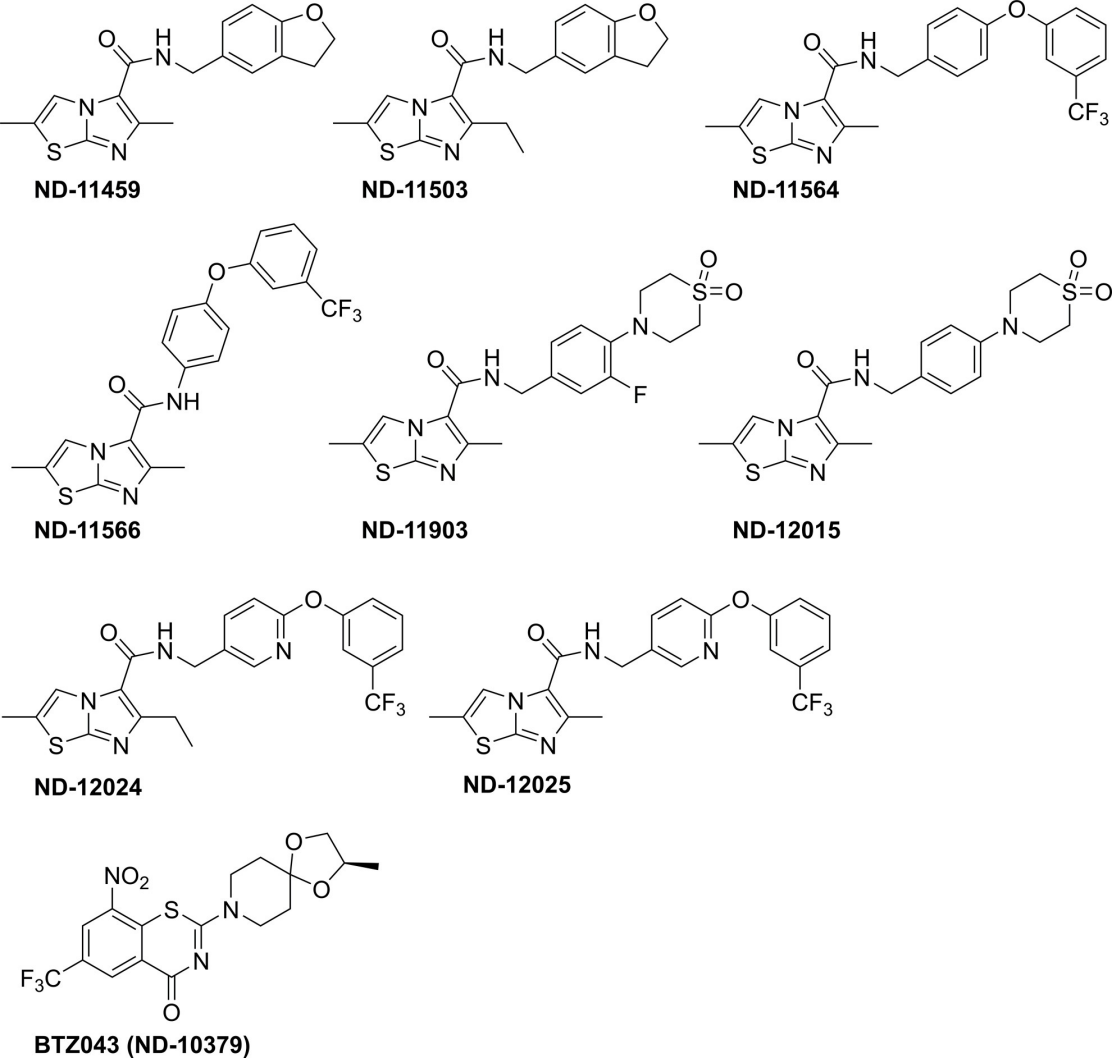
Additional eight Imidazo[2,1-b]thiazole-5-carboxamides and clinical candidate BTZ043 evaluated in this study.

Nine imidazo[2,1-*b*]thiazole-5-carboxamides were prepared and evaluated in this study (ND-11543, Figs 1 and 3) along with clinical candidates Q203 [16] (Fig 1) and BTZ043 [40] (Fig 3) as assay controls.

The *in vitro* potency against replicating H37Rv-Mtb, non-replicating H37Rv-Mtb (LORA), toxicity to mammalian cells (VERO) and aqueous solubility were determined for these compounds and a positive control, Q203 (Table 1). The ITA class showed very similar SAR trends to those observed with very structurally similar imidazo[1,2-*a*]pyridine-3-carboxamines [9,10,15,17,18] and pyrazolo[1,5-*a*]pyridine-3-carboxamides [29–31]. For instance, the most potent compounds were often the most lipophilic compounds as exemplified by ND-11543 and ND-11564 (clogP of 5.4 and 6.6, respectively) whereas polar compounds like ND-11903 and ND-12015 (clogP of 2.4 and 2.1, respectively) were >100-fold less active by comparison (Table 1). Benzylic amides were superior to anilides as demonstrated by ND-11564 and ND-11566 having a >50-fold difference in potency (MIC = 0.160 μM vs. 9.1 μM). Two compounds (ND-11459 and ND-11503) obey the “rule of five” [41] rubric and had good potency (MIC = 0.25–0.54 μM) and high aqueous solubility ≥200 μM at pH 6.8 (Table 1). All compounds showed greatly diminished activity in anerobic, non-replicating conditions (MIC ranging from 1.1 to 200 μM). This could be due to the mechanism of action of these compounds, targeting inhibition of cytochrome C oxidase [26] and the respiratory flexibility of that target [25]. All compounds showed no cellular toxicity (IC_50_ >50 μM).

**Table 1:** *In vitro* evaluation of nine imidazo[2,1-*b*]thiazole-5-carboxamides, Q203 and BTZ043 against replicating and non-replicating Mtb, cellular toxicity and aqueous solubility determination.

Cmpd ID	Mol Wt	Clog P	Aqueous solubility (μM)	MIC H37Rv-Mtb (μM)	LORA MIC (μM)	VERO Toxicity IC_50_ (μM)
**ND-11459**	327.40	3.63	>200	0.54	12.4	>50
**ND-11503**	341.43	4.16	>200	0.25	2.70	>50
**ND-11543**	532.56	5.43	50	0.008	1.10	>50
**ND-11564**	445.46	6.56	100	0.16	9.68	>50
**ND-11566**	431.43	6.38	50	9.10	>100	>50
**ND-11903**	436.52	2.38	>200	3.70	200	>50
**ND-12015**	418.53	2.07	200	13.0	ND*	>50
**ND-12024**	460.47	5.59	50	0.21	ND	>50
**ND-12025**	446.45	5.06	100	0.33	ND	>50
**Q203** **(ND-11496)**	557.01	7.62	100	0.005	1.3	>50
**BTZ043** **(ND-10379)**	431.39	2.45	ND	0.002	ND	>50

*Not Determined

### ITAs are potent against intracellular *M*. *tuberculosis*

**Fig 4:**
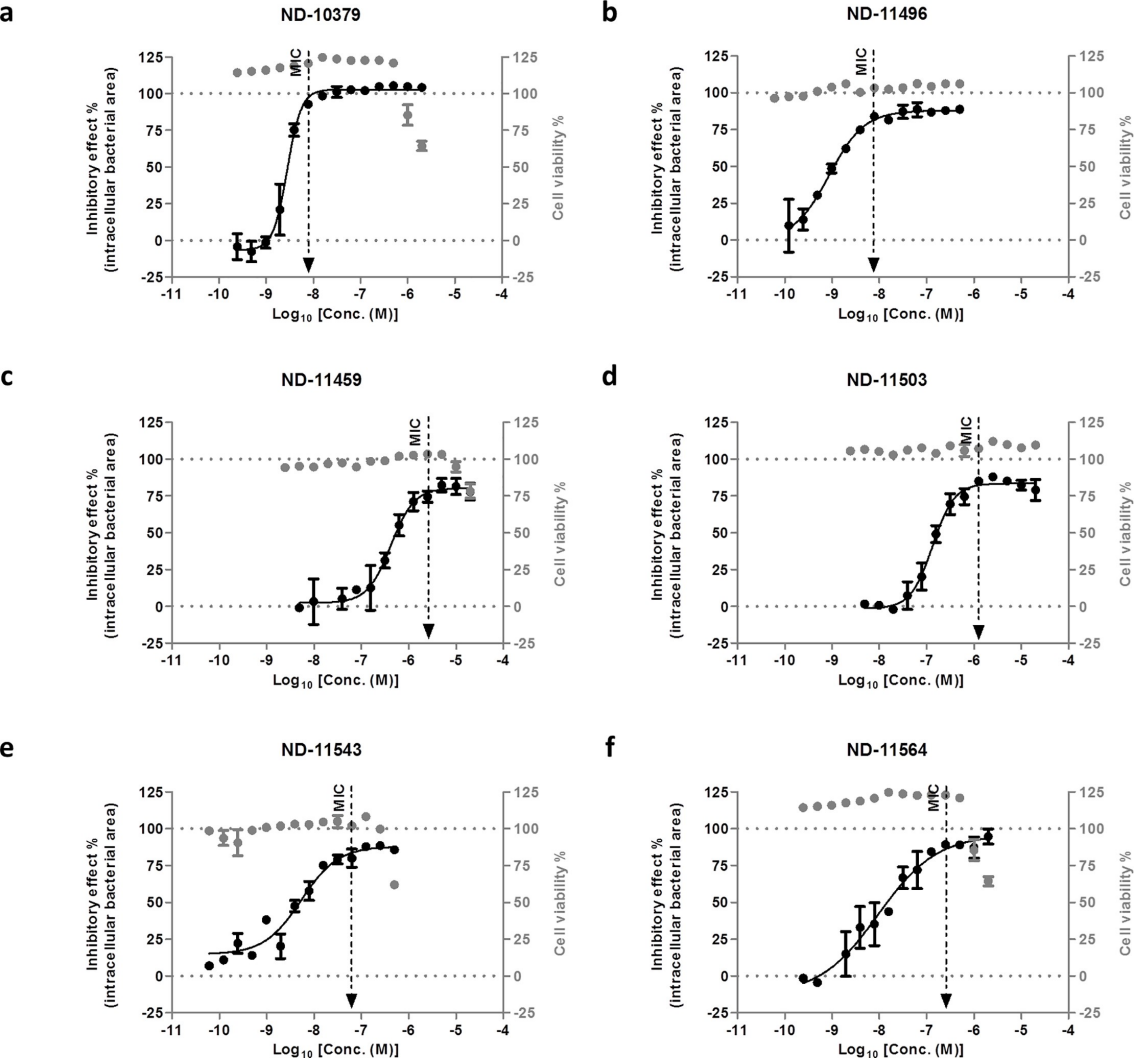
Effect on bacterial replication in murine raw 264.7 macrophages and on cell viability of (c) compound ND-11459, (d) compound ND-11503, (e) compound ND-11543 and (f) compound ND-11564 in comparison to two positive controls (a) BTZ043 (ND-10379) and (b) Q203 (ND-11496). The parameters used as a read-out were the area of bacteria present per infected macrophage and the total cell number, used as a cytotoxicity or viability control. Percentages of inhibitory effect were normalized based on the average values obtained for the negative control DMSO 1% (0% inhibition) and the positive control INH (isoniazid) 10 μg/mL (100% inhibition). Cell viability was measured by comparison of the cell number obtained for each compound concentration to the average value obtained for the positive control (isoniazid 10 μg/mL). Percentage of inhibitory effect and cell viability were plotted against the log10 of the compound concentrations. Fitting was performed for percentage of inhibition by Prism software using the sigmoidal dose−response (variable slope) model. The minimum inhibitory concentration (MIC) was determined and corresponded to the first concentration on the top of the curve, corresponding to the maximum inhibition. Experiments were carried out in duplicate.

The ability of compounds to inhibit *M*. *tuberculosis ex vivo* is a desirable characteristic. We tested a focused set of four ITAs to determine their activity in an intracellular model of *M*. *tuberculosis* infection along with two positive controls BTZ043 (ND-10379) and Q203 (ND-11496). Experimental data from the intracellular *M*. *tuberculosis* growth assay are shown in Fig 4. As anticipated, the positive controls BTZ043 and Q203 displayed very potent activity as reflected by an MIC of 0.0078 μM for each in these experiments (Fig 4(A) and 4(B), respectively) which is consistent with previous reports of about <0.098 μM [27]. The four selected ITA compounds were active against *M*. *tuberculosis* in the macrophage assay. ND-11543 and 11564 (Fig 4(E) and 4(F); respectively) were shown to exhibit lower MIC values of 0.0625 and 0.25 μM, respectively, than those obtained for the 2 other compounds (ND-11503 and 11459) ranging from 1.25–2.5 μM (Fig 4(D) and 4(C); respectively). All compounds were devoid of cytotoxicity on murine Raw 264.7 macrophages. Effect on the cell viability was shown for ND-11564, ND-11459 and ND-11543 which, respectively, showed macrophage toxicity at concentrations 4 and 8-fold higher than their MIC. These experiments showed that imidazo[2,1-*b*]thiazole-5-carboxamide compounds are active against pathogens residing intracellularly in macrophages, particularly against *Mycobacterium tuberculosis*, and highlight two more potent ITA compounds, ND-11543 and 11564.

### ITAs are potent against drug resistant strains

With the rapid rise in drug resistant TB, finding new compounds which can potentially inhibit strains resistant to first line drugs is of great interest. To that end, we screened four compounds and four positive controls (Q203, isoniazid, rifampicin, and levoflaxacin) against a panel of five drug resistant strains (Table 2). Screening reported here revealed that even moderately active compounds like ND-11903 and ND-11529 remain active even against hard to kill drug resistant strains and highlights the low nanomolar potency of ND-11543 particularly against RIF and INH resistant strains.

**Table 2:** Potency of four ITAs and Q203 against isoniazid, rifampicin and fluoroquinolone drug resistant Mtb strains.

	MIC (μM)				
Compd	FQ res^[a]^	INH res1^[b]^	INH res2^[c]^	RIF res1^[d]^	RIF res2^[e]^
**ND-11529**	0.93	1.2	0.64	0.75	1.0
**ND-11543**	0.010	0.012	0.0047	0.0017	0.0057
**ND-11903**	3.2	6.5	5.0	2.9	7.0
**ND-12024**	0.40	0.49	0.22	0.047	0.13
**Q203**	0.0058	0.0074	0.0038	0.0030	0.0056
**INH**^**[f]**^	0.34	>200	>200	0.160	0.370
**RIF**^**[g]**^	0.043	0.027	0.013	3.2	>50
**LEV**^**[h]**^	91	3.97	5.83	3.43	3.83

[a] Fluoroquinolone resistant strain derived from H37Rv Mtb and has a *gyrA* mutant (D94N)

[b] Isoniazid resistant strain derived from H37Rv Mtb and has a *katG* mutant (Y155* = truncation)

[c] Isoniazid resistant strain ACTCC35822

[d] Rifampicin resistant strain derived from H37Rv Mtb and is a *ropB* mutant (S522L)

[e] Rifampicin resistant strain ACTCC35838

[f] Isoniazid

[g] Rifampicin

[h] Levoflaxacin.

#### *In vitro* ADME of ND-11543

ND-11543 was a good candidate for ADME evaluation based upon its impressive *in vitro* potency against replicating and drug-resistant Mtb. Table 3 relates the plasma protein binding, Caco-2 permeability, cytochrome P450 inhibition, and human microsomal stability of ND-11543. ND-11543 was completely protein bound (100%) consistent with compounds with higher clogP values like Q203 [27]. Caco-2 values give an approximation of compound absorption and compounds with an efflux ratio of greater than 2 might be subject to active efflux. However, this is not expected to be an issue for ND-11543 as talinolol (a known P-glycoprotein substrate with blood brain transport) has an efflux ratio of 93.9 in this assay. Another desired characteristic of new TB therapeutics would be to have low drug-drug interactions. ND-11543 has high IC_50_ values ranging from >10 μM to > 20 μM against all the major cytochrome P450 isoforms coupled with low MIC values against H37Rv Mtb (Table 3). Lastly, ND-11543 demonstrated moderate stability with a half-life of 28.4 min in human liver microsomes.

**Table 3:** In vitro ADME evaluation of ND-11543.

	ND-11543
**Plasma Fraction Bound**^[a]^ (Mean %)	100
**Caco-2**^[b]^ P_app_ (10^−6^ cm/s)	
Mean A–B (1h)	0.376
Mean B–A (1h)	1.09
Mean A–B (2h)	0.731
Mean B–A (2h)	0.758
Efflux Ratio (average)	2.23
**Cytochrome P450 Inhibition** IC_50_ (μM)	
CYP2B6	>20
CYP2C8	18.4
CYP2C9	10.2
CYP2C19	>20
CYP2D6	>20
CYP3A4^[c]^	>20
CYP3A4^[d]^	>20
**Human liver S9 Microsomes**	
CL (μL/min/mg)	81.3
T ½ (min)	28.4

[a] Plasma protein binding for each test compound wsa determined by equilibrium dialysis

[b] Permeability using a Caco-2 cell monolayer in both directions. For A-B permeability, test compound was added to the apical side of the Caco-2 monolayer and the transport of compound to the basal side monitored. For B-A permeability, test compound iwas added to the basal side of the Caco-2 monolayer and the transport of the compound to the apical side monitored. Assays were run for 2h in duplicate.

[c] CYP3A4 drug-drug interaction (DDI) assay including midazolam [42]

[d] CYP3A4 DDI assay including tetosterone [42].

#### *In vivo* tolerability of ND-11543

Based upon its encouraging *in vitro* ADME properties, ND-11543 was assessed in an acute safety and tolerability mouse model to determine its Maximum Tolerated Dose, MTD. No acute toxicity was observed resulting in an MTD of >500 mg/kg.

#### *In vivo* PK of ND-11543

Based on MIC and acute toxicity data, ND-11543 was selected for evaluation in a single dose pharmacokinetic study with and without pre-dosing of a CYP-inhibitor (ABT) in Balb/c mice (Table 4). ND-11543 had reasonable drug exposure (AUC = 11,704 ng·hr/mL over 24 hr) and a Cmax (645 ng/mL) that remained above the MIC for 24 hr. The absolute AUC could not be calculated due to drug being present after 24 hr. When ABT was added, with a three-fold increased drug exposure (AUC = 37,250 ng·hr/mL over 24 hr) and Cmax (2,411 ng/mL). The large shift in tmax in presence of ABT (from 2 to 24 hr) resulted in a long half-life. This compound appeared to be slowly absorbed in presence of ABT as drug levels increased over 24 hr.

**Table 4:** Pharmacokinetics evaluation of 200 mg/kg ND-11543 with and without 100 mg/kg ABT.

pK parameters	ND-11543	ND-11543 + ABT
AUC(0–24) (ng·hr/mL)	11,704	37,250
AUC(0–24)/Dose (ng·hr/mL/mg/kg)	58.5	186
AUC(0-∞) (ng·hr/mL)	NC*	NC*
tmax (hr)	2	24
Cmax (ng/mL)	645	2,411
Cmax/Dose (ng/mL/mg/kg)	3.22	12.1
t1/2 (hr)	>24*	>24*
Relative Bioavailability [(TA+ABT) / TA]*	318.25	

*NC Not Calculable, the slope of the termination phase was positive.

#### ND-11543 is active in a chronic model of TB

ND-11543 was evaluated in a chronic Balb/c murine model of TB at either 100 or 200 mg/kg oral dose with or without ABT (Tables 5 and 6). The dose of 200 mg/kg ND-11543 with ABT was selected as this dose results in similar plasma drug exposure over 24 hr compared to Q203 (AUC 0–24 = 37,250 ng·hr/mL vs. 44,100 ng·hr/mL; respectively) [16]. As observed with other QcrB inhibitors, Q203 and TB47, ND-11543 was found to be active in the chronic murine model [30].

**Table 5:** *In vivo* murine efficacy of 200 mg/kg ND-11543 dosed for four weeks in Erdman infected mice with and without ABT.

Group (dose mg/kg)	Lung Log10 CFU ±SEM	Log reduction vs Control (Lung)	*n	Spleen Log10 CFU ±SEM	Log reduction vs Control (Spleen)	*n
Day 21 Pretreatment Control	5.87 ± 0.10		5/5	4.35 ± 0.18		5/5
**4 Week Treatment**						
ND-011543 (200)	5.49 ± 0.08	0.30	6/6	4.64 ± 0.11	0.36	6/6
ND-011543 (200) + ABT (100)	5.20 ± 0.22	0.59	4/5	4.23 ± 0.16	0.77	4/5
Control	5.79 ± 0.12		6/6	5.00 ± 0.13		6/6
INH (25)	4.06 ± 0.11	1.73	6/6	2.13 ± 0.12	2.87	5/6

*n = number of surviving mice.

**Table 6:** In vivo murine efficacy of 100 mg/kg ND-11543 dosed for four weeks in Erdman infected mice with 100 mg/kg ABT.

Group (dose mg/kg)	Lung Log10 CFU ±SEM	Log reduction vs CTL (Lung)	*n	Log10 CFU ±SEM	Log reduction vs CTL (Spleen)	*n
Day 35 Pretreatment Control	5.60 ± 0.08		5/5	4.59 ± 0.09		5/5
**4 Week Treatment**						
ND-011543 (100) + ABT (100)	5.11 ± 0.04	0.29	6/6	4.26 ± 0.11	0.82	6/6
Control	5.40 ± 0.09		5/5	5.08 ± 0.11		5/5

*n = number of surviving mice.

Mice treated with 200 mg/kg of ND-11543 showed a slight reduction in CFU in both lung and spleen (0.30 and 0.36 log10 CFU, respectively), but this was not statistically significant relative to control (Table 5) (P > 0.05). However, ND-11543 (200 mg/kg) + ABT (100 mg/kg) treatment resulted in a 0.59 log10 CFU reduction in the lung, and a 0.77 log10 CFU reduction in the spleen, both of which were statistically significant (p = 0.03, p<0.001 in lung and spleen, respectively). Two animals in this group showed adverse effects late in the first week of dosing. One animal was euthanized and the other animal, which showed minor lethargy, recovered. There were no other adverse effects noted in this treatment group over the course of the study. Due to the slight adverse effects at 200 mg/kg, ND-11543 (100 mg/kg) + ABT (100 mg/kg) treatment was evaluated in a follow up study (Table 6). This treatment resulted in a 0.29 log10 CFU reduction in the lung, and a 0.82 log10 CFU reduction in the spleen, both of which were statistically significant (p = 0.03, p<0.001 in lung and spleen, respectively).

## Conclusions

Nine imidazothiazoles (ITAs) were prepared and evaluated for their *in vitro* and *in vivo* properties revealing many potent compounds against replicating, drug resistant and intracellular Mtb. ND-11543 displayed good *in vivo* pharmacokinetics and tolerability. To the best of our knowledge, this is the first disclosure of an ITA that could halt TB disease progression in the chronic murine model of TB infection. Recent literature has shown that other QcrB inhibitors (namely, Q203 and TB47) have better efficacy within the acute model of TB infection and when used in combination with first line drugs such as rifampicin and pyrazinamide [30]. Due to the respiratory flexibility of Mtb there have been many reports that the efficacy of QcrB inhibitors might be greatly enhanced by tandem inhibition of the cytochrome bd oxidase based upon *in vitro* data [21, 22, 43–45]. However, this dual inhibition concept was strengthened by the impressive *in vivo* efficacy observed when QcrB inhibitors were evaluated against *Mycobacterium ulcerans*, a disease-causing strain which lacking cyt-bd oxidase [46–48]. Overall, ND-11543 presents a proof of principle compound which sets a new foundation for the design of TB drugs based on the imidazo[2,1-*b*]thiazole-5-carboxamide class.

## Supporting information

S1 Table Analysis sequence using Columbus system (version 2.3.1, PerkinElmer). (DOCX)

## References

[1] WHO (2018) Global Tuberculosis Report 2018. Geneva, Switzerland: WHO

[2] Sotgiu G, Centis R, D'ambrosio L, Migliori GB (2015) Tuberculosis treatment and drug regimens. Cold Spring Harb Perspect Med 5: a017822 10.1101/cshperspect.a017822 25573773 PMC4448591

[3] Sahu S, Ditiu L, Zumla A (2019) After the UNGA High-Level Meeting on Tuberculosis—what next and how? The Lancet Global Health 7: e558–e560. 10.1016/S2214-109X(19)30068-3 30876836

[4] Mahajan R (2013) Bedaquiline: first FDA-approved tuberculosis drug in 40 years. Int J Appl Basic Med Res 3: 1–2. 10.4103/2229-516X.112228 23776831 PMC3678673

[5] Pontali E, Raviglione MC, Migliori GB and the writing group members of the Global TB Network Clinical Trials Committee (2019) Regimens to treat multidrug-resistant tuberculosis: past, present and future perspectives. Eur Respir Rev 30: 190035. 10.1183/16000617.0035-2019 PMC9489134 31142549

[6] Maxmen A (2019) Treatment for extreme drug-resistant tuberculosis wins US government approval. Nature 10.1038/d41586-019-02464-0 32788696

[7] Mahajan R (2013) Bedaquiline: first FDA-approved tuberculosis drug in 40 years. Int J Appl Basic Med Res 3: 1–2. 10.4103/2229-516X.112228 23776831 PMC3678673

[8] Rao SP, Alonso S, Rand L, Dick T, Pethe K (2008) The protonmotive force is required for maintaining ATP homeostasis and viability of hypoxic, nonreplicating *Mycobacterium tuberculosis*. Proc Natl Acad Sci USA 105: 11945–11950. 10.1073/pnas.0711697105 18697942 PMC2575262

[9] Moraski GC, Markley LD, Hipskind PA, Boshoff H, Cho S, et al (2011) Advent of Imidazo[1,2-*a*]pyridine-3-carboxamides with Potent Multi- and Extended Drug Resistant Antituberculosis Activity. ACS Med Chem Lett 2: 466–470. 10.1021/ml200036r 21691438 PMC3117668

[10] Moraski GC, Markley LD, Cramer J, Hipskind PA, Boshoff H, et al (2013) Advancement of Imidazo[1,2-*a*]pyridines with Improved Pharmacokinetics and Nanomolar Activity Against *Mycobacterium tuberculosis*. ACS Med Chem Lett 4: 675–679. 10.1021/ml400088y 23930153 PMC3733398

[11] Moraski GC, Oliver AG, Markley LD, Cho S, Franzblau SG, et al (2014) Scaffold-switching: an exploration of 5,6-fused bicyclic heteroaromatics systems to afford antituberculosis activity akin to the imidazo[1,2-*a*]pyridine-3-carboxylates. Bioorg Med Chem Lett 24: 3493–3498. 10.1016/j.bmcl.2014.05.062 24909079 PMC4096046

[12] Cheng Y, Moraski GC, Cramer J, Miller MJ, Schorey JS (2014) Bactericidal activity of an imidazo[1,2-*a*]pyridine using a mouse *M*. *tuberculosis* infection model. PLoS One 9: e87483 10.1371/journal.pone.0087483 24498115 PMC3909116

[13] Moraski GC, Miller PA, Bailey MA, Ollinger J, Parish T, et al (2015) Putting Tuberculosis (TB) To Rest: Transformation of the Sleep Aid, Ambien, and "Anagrams" Generated Potent Antituberculosis Agents. ACS Infect Dis 1: 85–90. 10.1021/id500008t 25984566 PMC4426345

[14] Moraski GC, Cheng Y, Cho S, Cramer JW, Godfrey A, et al (2016) Imidazo[1,2-*a*]Pyridine-3-Carboxamides Are Active Antimicrobial Agents against *Mycobacterium avium* Infection *In Vivo*. Antimicrob Agents Chemother 60: 5018–5022. 10.1128/AAC.00618-16 27216051 PMC4958206

[15] Ollinger J, Bailey MA, Moraski GC, Casey A, Florio S, et al (2013) A dual read-out assay to evaluate the potency of compounds active against *Mycobacterium tuberculosis*. PLoS One 8: e60531 10.1371/journal.pone.0060531 23593234 PMC3617142

[16] Pethe K, Bifani P, Jang J, Kang S, Park S, et al (2013) Discovery of Q203, a potent clinical candidate for the treatment of tuberculosis. Nat Med 19: 1157–1160. 10.1038/nm.3262 23913123

[17] Kang S, Kim RY, Seo MJ, Lee S, Kim YM, et al (2014) Lead optimization of a novel series of imidazo[1,2-*a*]pyridine amides leading to a clinical candidate (Q203) as a multi- and extensively-drug-resistant anti-tuberculosis agent. J Med Chem 57: 5293–5305. 10.1021/jm5003606 24870926

[18] Abrahams KA, Cox JA, Spivey VL, Loman NJ, Pallen MJ, et al (2012) Identification of novel imidazo[1,2-*a*]pyridine inhibitors targeting *M*. *tuberculosis* QcrB. PLoS One 7: e52951 10.1371/journal.pone.0052951 23300833 PMC3534098

[19] O'Malley T, Alling T, Early JV, Wescott HA, Kumar A, et al (2018) Imidazopyridine compounds inhibit mycobacterial growth by depleting ATP levels. Antimicrob Agents Chemother 62: e02439–17. 10.1128/AAC.02439-17 29632008 PMC5971599

[20] Rybniker J, Vocat A, Sala C, Busso P, Pojer F, et al (2015) Lansoprazole is an antituberculous prodrug targeting cytochrome bc1. Nature Comm 6: 7659. 10.1038/ncomms8659 PMC4510652 26158909

[21] Foo CS, Lupien A, Kienle M, Vocat A, Benjak A, et al (2018) Arylvinylpiperazine Amides, a New Class of Potent Inhibitors Targeting QcrB of *Mycobacterium tuberculosis*. Mbio. 9: e01276–18. 10.1128/mBio.01276-18 30301850 PMC6178619

[22] Cleghorn LA, Ray PC, Odingo J, Kumar A, Wescott H, et al (2018) Identification of morpholino thiophenes as novel *Mycobacterium tuberculosis* inhibitors, targeting QcrB. J Med Chem 61: 6592–6608. 10.1021/acs.jmedchem.8b00172 29944372 PMC6089501

[23] Berube BJ, Parish T (2018) Combinations of respiratory chain inhibitors have enhanced bactericidal activity against *Mycobacterium tuberculosis*. Antimicrob Agents Chemother 62: e01677–17. 10.1128/AAC.01677-17 29061760 PMC5740367

[24] Chandrasekera NS, Berube BJ, Shetye G, Chettiar S, O’Malley T, et al (2017) Improved phenoxyalkylbenzimidazoles with activity against *Mycobacterium tuberculosis* appear to target QcrB. ACS Infect Dis 3: 898–916. 10.1021/acsinfecdis.7b00112 29035551 PMC5727484

[25] Arora K, Ochoa-Montaño B, Tsang PS, Blundell TL, Dawes SS, et al (2014) Respiratory flexibility in response to inhibition of cytochrome C oxidase in *Mycobacterium tuberculosis*. Antimicrob Agents Chemother 58: 6962–6965. 10.1128/AAC.03486-14 25155596 PMC4249445

[26] Moraski GC, Seeger N, Miller PA, Oliver AG, Boshoff HI, et al (2016) Arrival of Imidazo[2,1-*b*]thiazole-5-carboxamides: Potent Anti-tuberculosis Agents That Target QcrB. ACS Infect Dis 2: 393–398. 10.1021/acsinfecdis.5b00154 27627627

[27] Moraski GC, Bristol R, Seeger N, Boshoff HI, Tsang PS, et al (2017) Preparation and Evaluation of Potent Pentafluorosulfanyl‐Substituted Anti‐Tuberculosis Compounds. ChemMedChem 12: 1108–1115. 10.1002/cmdc.201700170 28654200 PMC5603227

[28] Lu X, Tang J, Liu Z, Li M, Zhang T, et al (2016) Discovery of new chemical entities as potential leads against *Mycobacterium tuberculosis*. Bioorg Med Chem Lett 26: 5916–5919. 10.1016/j.bmcl.2016.11.003 27839917

[29] Tang J, Wang B, Wu T, Wan J, Tu Z, et al (2015) Design, synthesis, and biological evaluation of pyrazolo[1,5-*a*]pyridine-3-carboxamides as novel antitubercular agents. ACS Med Chem Lett 6: 814–818. 10.1021/acsmedchemlett.5b00176 26191372 PMC4499832

[30] Lu X, Williams Z, Hards K, Tang J, Cheung CY, et al (2018) Pyrazolo[1,5-*a*]pyridine Inhibitor of the Respiratory Cytochrome bcc Complex for the Treatment of Drug-Resistant Tuberculosis. ACS Infect Dis 5: 239–249. 10.1021/acsinfecdis.8b00225 30485737

[31] Hu X, Wan B, Liu Y, Shen J, Franzblau SG, et al (2019) Identification of Pyrazolo[1,5-*a*]pyridine-3-carboxamide Diaryl Derivatives as Drug Resistant Anti-tuberculosis Agents. ACS Med Chem Lett 10: 295–299 10.1021/acsmedchemlett.8b00410 30891129 PMC6421536

[32] Queval CJ, Song OR, Delorme V, Iantomasi R, Veyron-Churlet R, et al (2014) A microscopic phenotypic assay for the quantification of intracellular mycobacteria adapted for high-throughput/high-content screening. J Vis Exp 83: e51114. 10.3791/51114 PMC4089478 24473237

[33] Song OR, Deboosere N, Delorme V, Queval CJ, Deloison G, et al (2017) Phenotypic assays for *Mycobacterium tuberculosis* infection. *Cytometry A* 91: 983–994. 10.1002/cyto.a.23129 28544095

[34] Sprouffske K, Wagner A (2016) Growthcurver: an R package for obtaining interpretable metrics from microbial growth curves. BMC Bioinformatics 17: 172 10.1186/s12859-016-1016-7 27094401 PMC4837600

[35] Cho SH, Warit S, Wan B, Hwang CH, Pauli GF, et al (2007) Low-Oxygen-Recovery Assay for high-throughput screening of compounds against nonreplicating *Mycobacterium tuberculosis*. Antimicrob Agents Chemother 51: 1380–1385. 10.1128/AAC.00055-06 17210775 PMC1855511

[36] Wayne LG, Hayes L (1998) Nitrate reduction as a marker for hypoxic shiftdown of *Mycobacterium tuberculosis*. Tuber Lung Dis. 79: 127–132. 10.1054/tuld.1998.0015 10645451

[37] Falzari K, Zhu Z, Pan D, Liu H, Hongmanee P, et al (2005) In vitro and in vivo activities of macrolide derivatives against *Mycobacterium tuberculosis*. Antimicrob Agents Chemother 49: 1447–1454. 10.1128/AAC.49.4.1447-1454.2005 15793125 PMC1068601

[38] Brodin P, Poquet Y, Levillain F, Peguillet I, Larrouy-Maumus G, et al (2010) High content phenotypic cell-based visual screen identifies Mycobacterium tuberculosis acyltrehalose-containing glycolipids involved in phagosome remodeling. PLoS Pathog 6: e1001100 10.1371/journal.ppat.1001100 20844580 PMC2936551

[39] Lenaerts AJ, Gruppo V, Marietta KS, Johnson CM, Driscoll DK, et al (2005) Preclinical testing of the nitroimidazopyran PA-824 for activity against *M*. *tuberculosis* in a series of *in vitro* and *in vivo* models. Antimicrob Agents Chemother 49: 2294–2301. 10.1128/AAC.49.6.2294-2301.2005 15917524 PMC1140539

[40] Tiwari R, Moraski GC, Krchňák V, Miller PA, Colon-Martinez M, et al (2013) Thiolates chemically induce redox activation of BTZ043 and related potent nitroaromatic anti-tuberculosis agents. J Am Chem Soc 135: 3539–3549. 10.1021/ja311058q 23402278 PMC3677520

[41] Lipinski CA (2004) Lead-and drug-like compounds: the rule-of-five revolution. Drug Discov Today Technol 1: 337–341. 10.1016/j.ddtec.2004.11.007 24981612

[42] Foti RS, Rock DA, Wienkers LC, Wahlstrom JL (2010) Selection of alternative CYP3A4 probe substrates for clinical drug interaction studies using *in vitro* data *and in vivo* simulation. Drug Metab Dispos 38: 981–987. 10.1124/dmd.110.032094 20203109

[43] Cook GM, Hards K, Dunn E, Heikal A, Nakatani Y, et al (2017) Oxidative Phosphorylation as a Target Space for Tuberculosis: Success, Caution, and Future Direction. Microbiol Spectr 5: 10.1128/microbiolspec TBTB2-0014-2016. PMC5480969 28597820

[44] Kalia NP, Hasenoehrl EJ, Ab Rahman NB, Koh VH, Ang ML, et al (2017) Exploiting the synthetic lethality between terminal respiratory oxidases to kill *Mycobacterium tuberculosis* and clear host infection. Proc Natl Acad Sci USA 114: 7426–7431. 10.1073/pnas.1706139114 28652330 PMC5514758

[45] Lu P, Asseri AH, Kremer M, Maaskant J, Ummels R, et al (2018) The anti-mycobacterial activity of the cytochrome bcc inhibitor Q203 can be enhanced by small-molecule inhibition of cytochrome bd. Sci Rep 8: 2625 10.1038/s41598-018-20989-8 29422632 PMC5805707

[46] Scherr N, Bieri R, Thomas SS, Chauffour A, Kalia NP, et al (2018) Targeting the *Mycobacterium ulcerans* cytochrome bc1:aa3 for the treatment of Buruli ulcer. Nature Comm 9: 5370. 10.1038/s41467-018-07804-8 PMC6299076 30560872

[47] Liu Y, Gao Y, Liu J, Tan Y, Liu Z, et al (2019) The compound TB47 is highly bactericidal against *Mycobacterium ulcerans* in a Buruli ulcer mouse model. Nature Comm 10: 524. 10.1038/s41467-019-08464-y PMC6355801 30705268

[48] Converse PJ, Almeida DV, Tyagi S, Xu J, Nuermberger EL (2019) Shortening Buruli ulcer treatment with combination therapy targeting the respiratory chain and exploiting *M*. *ulcerans* gene decay. Antimicrob Agents Chemother 63: e00426–1. 10.1128/AAC.00426-19 31036687 PMC6591589

