# Supplementary material for: Intracellular and in vivo evaluation of imidazo[2,1-b]thiazole-5-carboxamide anti-tuberculosis compounds
Source: PLoS One. 2020 Jan 6;15(1):e0227224. doi: 10.1371/journal.pone.0227224 (PMC6944458; doi:10.1371/journal.pone.0227224)
Supplement: S1 Table — (DOCX) [file pone.0227224.s001.docx]

**Table S1.** Analysis sequence using **Columbus system (version 2.3.1, PerkinElmer).** An in-house multi-parameter script was developed for the analysis of images obtained for Raw 264.7 murine macrophages infected with *Mtb* strains.

| **Input Image** | **Stack Processing:** Individual Planes  **Flat field Correction:** None | **Method** | **Output** |
| --- | --- | --- | --- |
| **Calculate Image** |  | **Method :** By Formula  Formula : A-30  Channel A : Exp1Cam3  Negative Values : Set to Zero  Undefined Values : Set to Local Average | Output Image : Syto 60 - thresholded |
| **Find Nuclei** | **Channel :** Exp1Cam3  **ROI :** None | **Method :** C  Common Threshold : 0.5  Area : > 30 µm²  Split Factor : 7  Individual Threshold : 0.6  Contrast : > -0.05 | Output Population : Nuclei - preselected |
| **Calculate Intensity Properties** | **Channel :** Exp1Cam3  **Population :** Nuclei - preselected  **Region :** Nucleus | **Method :** Standard  Mean | Output Properties : Intensity Nucleus Exp1Cam3 |
| **Calculate Morphology Properties** | **Population :** Nuclei - preselected  **Region :** Nucleus | **Method :** Standard  Area  Roundness | Output Properties : Nucleus |
| **Select Population** | **Population :** Nuclei - preselected | **Method :** Filter by Property  Intensity Nucleus Exp1Cam3 Mean : > 100  Nucleus Area [µm²] : <= 250  Nucleus Roundness : > 0.5  Boolean Operations : F1 and F2 and F3 | Output Population : Nuclei |
| **Find Cytoplasm** | **Channel :** Syto 60 - thresholded  **Nuclei :** Nuclei | **Method :** A  Individual Threshold : 0.25 |  |
| **Select Population** | **Population :** Nuclei | **Method :** Common Filters  Remove Border Objects  Region : Cell | Output Population : Cells Selected |
| **Find Spots** | **Channel :** Exp1Cam2  **ROI :** Cells Selected | **Method :** D  Detection Sensitivity : 0.5  Splitting Coefficient : 0.5  Background Correction : 0.5  Calculate Spot Properties | Output Population : Spots |
| **Calculate Intensity Properties** | **Channel :** Exp1Cam2  **Population :** Spots  **Region :** Spot | **Method :** Standard  Mean | Output Properties : Intensity Spot Exp1Cam2 |
| **Select Population** | **Population :** Spots | **Method :** Filter by Property  Intensity Spot Exp1Cam2 Mean : > 190 | Output Population : Bacteria |
| **Calculate Morphology Properties** | **Population :** Bacteria  **Region :** Spot | **Method :** Standard  Area | Output Properties : Bacteria |
| **Calculate Properties** | **Population :** Cells Selected | **Method :** By Related Population  Related Population : Bacteria  Number of Bacteria  Bacteria Area [px²] | Output Properties : per Cell |
| **Select Population** | **Population :** Cells Selected | **Method :** Filter by Property  Number of Bacteria- per Cell : > 0 | Output Population : Infected Cells |
| **Define Results** | **Method:** List of Outputs  Number of Objects  **Population : Bacteria**  Bacteria Area [px²] : Sum  **Population : Infected Cells**  Bacteria Area [px²]- Sum per Cell : Mean  **Method :** Formula Output  Formula : a/b*100  Population Type : Objects  Variable A : Infected Cells - Number of Objects  Variable B : Cells Selected - Number of Objects  Output Name : % Infected cells | | |
